# Reasons for prescribing second generation antihistamines to treat allergic rhinitis in real-life conditions and patient response

**DOI:** 10.1186/1710-1492-10-29

**Published:** 2014-06-06

**Authors:** Pascal Demoly, Anca Mirela Chiriac, Benoît Berge, Michel Rostin

**Affiliations:** 1 Département de Pneumologie et Addictologie, Hôpital Arnaud de Villeneuve, University Hospital of Montpellier, France; and Sorbonne Universités, UPMC Paris 06, UMR-S 1136, IPLESP, Equipe EPAR, 75013 Paris, France; 2 EURAXI PHARMA, 10 rue Gutenberg, BP 80325, 37303 Joué-les-Tours, France; 3 Medical Affairs Department, Pierre Fabre Médicament, 81106 Castres Cedex, France

**Keywords:** Allergic rhinitis, Quality of life, Second generation antihistamine

## Abstract

**Background:**

Second generation H1 antihistamines (H1A) are currently recommended as first choice medications for allergic rhinitis and rhinoconjunctivitis. However, little is known about what influences the choice of prescription of one second generation (H1A) as opposed to another in real-life conditions.

**Objective:**

The aim of the study was to identify the main criteria determining the choice of a second generation H1A by allergy specialists in mainland France.

**Methods:**

Consecutive patients suffering from allergic rhinitis or rhinoconjunctivitis were included and followed prospectively for 30 days from the prescription of a second generation H1A in monotherapy. Patients were asked to fill in auto-questionnaires at baseline, daily during the first 10 days of the new treatment, and at the end of follow-up. Data on efficacy, tolerance, safety, rate and type of response to treatment, as well as patient satisfaction were recorded and analyzed.

**Results:**

1,080 patients were included between March 2011 and October 2012, mostly suffering from moderate to severe rhinitis (82.0%). The most frequently cited reason for choosing a specific H1A was the expected efficacy (85.3%). The mean time to nasal and ocular recovery was 6 days and 78.2% of patients responded to treatment within this interval. The presence of conjunctivitis was significantly associated with a more rapid response. At the end of follow-up, the satisfaction rate was higher for patients who were switched from a previous treatment (87.5%), compared to those receiving their first treatment (78.8%).

**Conclusion and clinical relevance:**

The main reason for choosing a specific second generation H1A was its expected efficacy. Concomitant conjunctivitis is associated with a more rapid response to treatment. Symptom recovery necessitates a mean of 6 days.

## Introduction

Allergic rhinitis remains one of the most prevalent diseases in Europe and constitutes an important health issue. In France, prevalence was assessed at about 30% in an adult population-based sample of more than 10,000 subjects [1]. Symptoms greatly impact general well-being and quality of life of a significant proportion of patients, particularly among those with persistent disease [2]. H1 antihistamine compounds (or H1A) are the main drugs prescribed to prevent or alleviate nasal and ocular symptoms of allergic rhinitis and conjunctivitis [3]. Because of the bothersome side effects (sedation, impaired cognitive and psychomotor functions) of the first marketed medications, second generation H1A have been developed and approved over the last 25 years. They exhibit a low sedating potential related to their poor ability to cross the blood–brain barrier. They are recommended as first choice medications for allergic rhinitis and conjunctivitis [4,5]. Up to date, 9 second generation H1A are available in France in various formulations. All have evidenced their efficacy and safety in randomized clinical trials. However, not enough data are available from studies in real-life conditions. Although most patients report being relieved by medication, one out of five remains unsatisfied, as shown by a self-completion survey conducted among European patients [6].

The present study was designed to examine, in current practice, the main reasons why physicians decide to change their patients’ treatment and identify the main criteria determining the choice of a second generation oral H1A. In addition, patient evaluation and satisfaction was assessed during the first month after treatment switch or initiation, in order to gather data on efficacy and tolerance in field conditions. Finally, the analysis attempted to describe the profile of patients who best responded to treatment.

## Methods

### Study design and setting

The study was a longitudinal, prospective, multicenter survey conducted in mainland France. Data collection did not interfere with patients’ medical care or with any of the physicians’ decisions. In accordance with French law, formal approval from an ethics committee is not required for observational studies. Before inclusion, the purpose and objectives of the study were explained to patients and written informed consent was obtained. All procedures were performed in accordance with the ethical principles of the Helsinki declaration, with Good Epidemiological Practice guidelines and with the national regulations in force. The French “Commission Nationale Informatique et Libertés”, an independent administrative body that operates in accordance with the data protection legislation, gave its approval for the study. Data were collected and managed anonymously.

### Participants

All French allergy specialists (i.e. 1,250 physicians) were invited to take part in the survey by mail. Assuming that 20% would accept, and 85% of them could actually include patients, the final pool was expected to include 215 physicians. Following their inclusion, the retained practitioners were to propose the survey to 10 consecutive patients meeting the inclusion criteria: (i) adult outpatient; (ii) diagnosed with allergic rhinitis, either intermittent or persistent (according to ARIA classification [7]), associated or not with allergic conjunctivitis, with no or mild and controlled asthma (according to GINA classification [8]); (iii) presenting with symptoms that require a second generation oral H1A in monotherapy, depending on the physician’s decision. It could be a treatment initiation, if no previous H1A therapy had been given or a switch from a previous H1A treatment, either first or second generation. Patients receiving any other treatment for allergic rhinitis (such as allergen immunotherapy) were excluded from the survey, as were pregnant or lactating women, or patients unable to answer auto-questionnaires.

### Data collection

Three different questionnaires were used during the survey. The first one (physician questionnaire) was filled in by the physician on the day of inclusion (D0) and contained items on socio-demographic characteristics, medical history of patients, previous treatment, new treatment chosen on the day of inclusion and the motivations for this choice. The second questionnaire (quality of life (QOL) auto-questionnaire), filled in on D0 and D30, focused on the description of symptoms and disorders related to allergic rhinitis and its treatments. Questions about satisfaction, perception of efficacy and tolerance of the second generation H1A were added to this questionnaire filled in on D30. Thirdly, a daily auto-questionnaire was filled in by the patient at home during the first 10 days of the new treatment (*i.e*. from D1 to D10). It assessed patient’s daily global, nasal and ocular symptoms (see below). On D10 (or on the last day of treatment, in the event of premature termination), questions about satisfaction, perception of efficacy and tolerance of the treatment were also added. Patients returned the auto-questionnaires by mail to the survey monitor at the end of follow-up.

### Objectives and outcomes

The primary assessment criterion was the main reason cited by physician for choosing a second generation oral H1A, as reported at inclusion (D0). Each choice was described for the whole cohort of subjects, or by subgroups, according to prior therapy, associated diagnosis of conjunctivitis or not, and the periodicity of symptoms (persistent or intermittent). The physician was asked to rank the three main reasons in a list: efficacy, rapidity of onset of action, length of action, global tolerance, cardiac safety, absence of sedative effect, absence of fatigue, absence of mouth dryness, pharmacokinetic characteristics, safety for at-risk patients, convenience of formulation. The main reason cited by the practitioner for giving up the previous treatment was also described (choices were: not convenient for at-risk patients, non-convenient formulation, insufficient efficacy, drug interaction issue, tolerance issue).

The secondary objectives were to assess the efficacy and tolerance of the newly prescribed second generation H1A in real-life conditions, and to characterize the profile of patients who best responded to this treatment. Alleviation of the main symptoms was reported in the 10-day auto-questionnaire. Patients rated daily three nasal symptoms (sneezing, congestion, rhinorrhea) and three ocular symptoms (itching eyes, painful eyes, tearing eyes) on a 4-point Likert scale graduated 0 = “no symptoms”; 1 = “mild and not troublesome symptoms”; 2 = “moderate, troublesome, but tolerable symptoms”; 3 = “severe, badly tolerated and perturbing symptoms”. A nasal score, an ocular score and a global symptom score were calculated each day as the sum of scores for the nasal symptoms, ocular symptoms, and all symptoms, respectively. Times to nasal recovery, ocular recovery and global recovery were determined when the nasal score, ocular score and global symptom score reached zero, respectively. A patient was considered as responding to treatment when the symptom score was reduced by 50% when compared to baseline, *i.e.* symptom score obtained at D1 before taking the first tablet of the new treatment (nasal, ocular and global response were characterized). The rate of responders was calculated daily between D2 and D10. A patient was classified as an early responder when he/she responded before the mean time to response of the whole cohort; as a late responder if he/she responded later; or as a non-responder if he/she did not respond until the 10^th^ day of treatment.

Patient satisfaction (rated on a 4-point Likert scale graduated from 0 = “not satisfied” until 3 = “very satisfied”) was described at D10 and D30, in the whole cohort and by previous treatment subgroups. Similarly, patients were also asked at D10 and D30 whether the new treatment was more effective, as effective, or less effective than the previous treatment to relieve their symptoms, whether it was better tolerated, equally well tolerated, or less well tolerated, and whether it was more rapid, as rapid, or less rapid to relieve their symptoms. Relief of disorders occurring during daily activities was assessed by the patient in the daily auto-questionnaire (from D1 to D10) using a numeric scale (0 = “no relief” until 10 = “total relief”). The effects of the new treatment on QOL (assessed by the Mini-RQLQ score [9] and a visual analog scale for global impact) and on daytime sleepiness (Epworth visual analog scale [10]) were described by the patient at D0 and D30. Furthermore, the cumulative distribution frequency of scores for mini-RQLQ was analyzed. The minimal clinically important difference (MCID) was set at 0.7 points, in accordance with recently published data [11].

The safety and tolerability were assessed by the occurrence of adverse events (AEs) throughout follow-up.

### Statistical methods

Patient characteristics were described for the whole cohort. Quantitative and qualitative variables were analyzed using usual descriptive statistics. The differences between groups of patients (rhinitis alone versus rhinoconjunctivitis; persistent versus intermittent disease) were explored through univariate and multivariate logistic regressions, entering sex, previous treatment, severity of the disease (according to ARIA classification [7]), time from allergic rhinitis onset, occurrence of asthma, occurrence of other allergies, familial history of allergy and response to treatment as explicative variables. Post-hoc analysis was performed to characterize the profile of responders (early versus late versus no response), through univariate and stepwise multivariate logistic regressions, entering the same variables plus occurrence of conjunctivitis and periodicity of disease. Univariate analyses were based on the Chi-2 test, and were entered in the stepwise model with a p value threshold of 0.35, then retained as significant for a 2-sided p value ≤ 0.05. Evolutions of numeric scores or visual analog scales throughout treatment were compared by the 2-sided Student T-test for matched measures.

The criterion of the primary objective (main reason for choosing the new second generation H1A) was reported with frequency and 95% confidence interval for the whole cohort, then for subgroups according to the occurrence of conjunctivitis (rhinitis alone versus rhinoconjunctivitis), and to the periodicity of the disease (persistent versus intermittent). Chi-2 test was used for comparing subgroups.

Data management and statistical analyses were performed using SAS 9.2 software.

### Sample size

Assuming that the adverse effects associated with first generation H1A treatment would prevail in about 30% of patients [12], that “good tolerance” was likely to be the most cited reason for choosing a second generation H1A, and assuming a standard deviation of 8%, taking a two-sided alpha risk of 5% and a rate of non-analyzable cases of 5%, 2,000 patients were required.

## Results

### Patients

Overall, 237 French allergy specialists participated in the study and included 1,080 patients between March 2011 and October 2012. Socio-demographic and medical characteristics of the patients are presented in Table 1.

**Table 1:** Socio-demographic and medical characteristics of the cohort

**Number of patients included**	**1,080**
Age* (n = 1080)	34.7 ± 12.7 years
Sex (n = 1046)	
Male	41.1%
Female	58.9%
Occupation (n = 1065)	
Employee	32.0%
Student	17.8%
Manager/intellectual professional	12.6%
Intermediate professional	9.8%
Other	27.8%
Time from allergic rhinitis onset* (n = 1034)	9.8 ± 9.7 years
Familial history of allergy (n = 1065)	56.4%
Periodicity of allergic rhinitis (n = 995)	
Persistent	63.2%
Intermittent	36.8%
Severity of symptoms (ARIA) (n = 884)	
Mild	18.0%
Moderate to severe	82.0%
Concomitant disease	
Conjunctivitis (n = 1070)	70.9%
Asthma (n = 1064)	26.0%
Other allergies (n = 1072)	16.6%
Atopic eczema (n = 1064)	6.4%
Sinusitis (n = 1064)	7.8%
Urticaria (n = 1064)	9.5%
Smoking (n = 1065)	
Active	16.3%
Passive	5.6%
Ceased	8.5%
No	69.6%
Regular contact with pet (n = 1048)	44.4%

*mean ± SD*.*

As shown by univariate analyses (Table 2), persistent rhinitis was more frequently associated with familial history of allergy, moderate to severe symptoms and asthma, whereas intermittent rhinitis was more frequently associated with conjunctivitis. According to multivariate analysis, the risk of a patient having asthma was 2.0 fold higher (95% confidence interval [1.2-3.3], p = 0.0069) if he/she experienced a persistent form of rhinitis, and the risk of having conjunctivitis symptoms was 1.8 fold higher (95% confidence interval [1.1-2.9], p = 0.0146) with the intermittent form. Other characteristics (sex, time from onset of allergy) were not significantly different between groups.

**Table 2:** Profile of patients with persistent or intermittent rhinitis at baseline

	**Intermittent rhinitis**	**Persistent rhinitis**	P value*
Frequency (n = 995)	36.5%	63.6%	
Familial history of allergy (n = 982)	55.4%	57.0%	0.652
Severity of symptoms (ARIA) (n = 837)			<0.001
Mild	24.8%	13.6%	
Moderate to severe	75.2%	86.4%	
Concomitant disease			
Conjunctivitis (n = 988)	74.5%	67.8%	0.0258
Asthma (n = 984)	22.2%	27.9%	0.0463

*2-sided Chi-2 test.

Similarly, patients suffering from rhinoconjunctivitis were more likely to exhibit moderate to severe symptoms than those with rhinitis alone (83.8% versus 77.6%; p = 0.03). Other characteristics were not significantly different between groups.

Overall, 12.2% of patients had not taken any treatment for their allergic rhinitis during the 24 months preceding inclusion. From the 87.8% who had been treated, 55.6% were insufficiently relieved by the treatments administered during the previous 24 months, as reported at inclusion by the physician. Among patients suffering from rhinoconjunctivitis, 73.7% took a specific treatment and 52.3% were insufficiently relieved. The therapeutic class of treatments administered in the previous 24 months were oral H1A (88.0% of patients), local H1A (12.5%), local corticosteroids (33.6%), cromones (8.7%) and leukotriene antagonists (6.8%).

### Reasons for treatment choice

**Figure 1:**
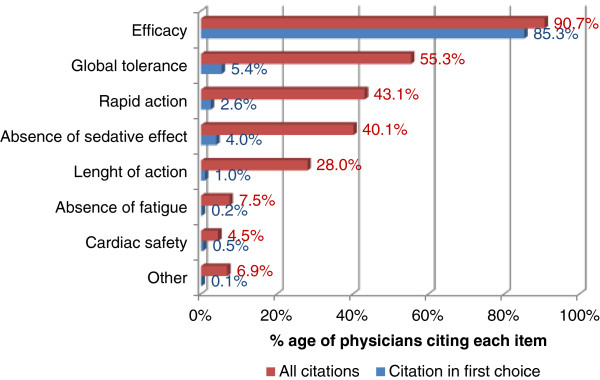
Reasons cited by physicians for selecting a second generation oral anti-H1.

On the day of inclusion, physicians prescribed a second generation oral H1A in monotherapy. The main molecule was bilastine (89.9% of cases), followed by ebastine (4.8%) and desloratadine (1.9%). The reasons cited to opt for this new treatment are shown in Figure 1. The most frequently cited reason was the expected efficacy, which was reported in 90.7% of cases (85.3% as the main reason).

Physicians also justified why they chose to stop the previous treatment. “Lack of efficacy” was the most cited reason (78.2% overall; 90.9% for ebastine; 88.0% for desloratadine), followed by “tolerance issue” (16.1% overall; 29.2% for mizolastine; 22.4% for levocetirizine; 21.9% for cetirizine).

### Efficacy and tolerance of the second generation H1-antihistamine

**Figure 2:**
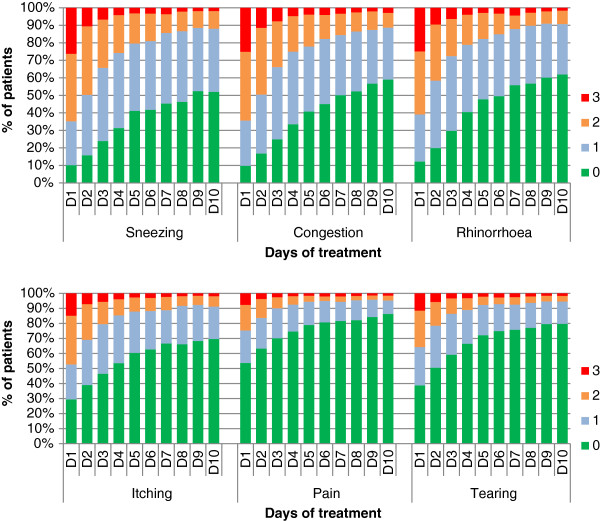
**Evolution of nasal and ocular symptoms between D1 and D10.** 0 = “no symptoms”; 1 = “mild and not troublesome symptoms”; 2 = “moderate, troublesome, but tolerable symptoms”; 3 = “severe, badly tolerated and perturbing symptoms.

**Figure 3:**
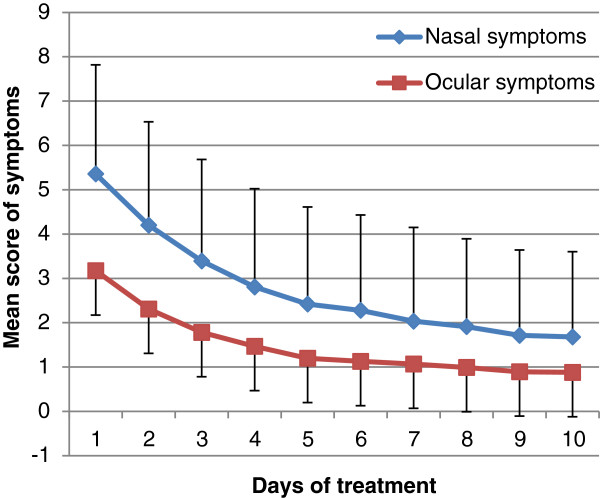
Mean scores of nasal and ocular symptoms between D1 and D10.

Symptoms were assessed daily during the 10 first days of treatment using a 4-point Likert scale. At D10, 719 out of 1,080 (*i.e.* 66.6%) returned the auto-questionnaire. Evolution of each symptom throughout time was very favorable, as shown in Figure 2 for nasal and ocular symptoms. Mean nasal and ocular symptom scores are shown in Figure 3.

At D30, at the end of follow-up, 685 out of 1,080 (*i.e.* 63.4%) returned the auto-questionnaire. Among them, 67.9% of patients considered that the new H1A was more effective than the previous treatment to relieve symptoms of allergic rhinitis; 26.2% as effective; and 5.9% less effective. They reported being very satisfied (47.2%) or satisfied (49%) with the new treatment. The satisfaction rate was higher (although no statistical test was performed) for patients who were switched from a previous treatment (87.5% satisfied or very satisfied), compared to the patients treated for the first time (78.8%). Patients who reported being satisfied had a mean improvement in nasal and ocular scores and in global mini-RQLQ score of 2.9, 2.4 and 1.4 points, respectively. Patients who were very satisfied with their treatment had mean score changes of 4.5, 3.0 and 2.1 points for nasal and ocular symptoms scores and global mini-RQLQ score, respectively.

**Figure 4:**
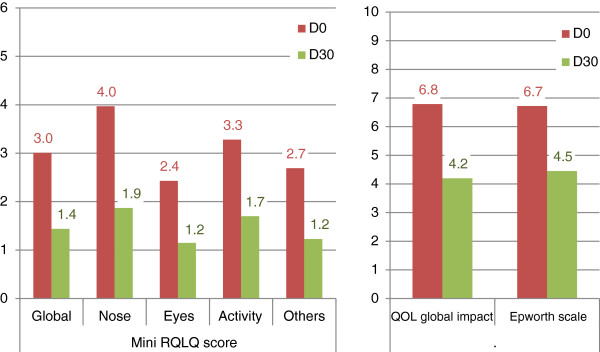
Evolution of QOL and daytime sleepiness between D0 and D30.

Patient QOL evolved favorably throughout follow-up, as assessed by the significant reduction in mini-RQLQ scores (global scores and sub-scores), measure of global impact using a visual analog scale and Epworth scale, between D0 and D30 (see Figure 4). Considering an anchor-based MCID of −0.7 points for mini-RQLQ [11]), and a distribution-based MCID of −1 point (*i.e.* half of standard deviation at baseline [11]) for the visual analog scale, a large majority of patients evidenced such improvements during treatment, as shown in Table 3. Ratios were similar whether patients had rhinitis alone or rhinoconjunctivitis, except for the eye mini-RQLQ sub-score.

**Table 3:** Change in quality of life and daytime sleepiness between D0 and D30

	**D30 – D0**	% of patients achieving MCID*		
	**Mean ± SD (N)**	**Whole cohort**	**Rhinitis alone**	**Rhinoconjunctivitis**
**Quality of life**				
**Mini-RQLQ score** (from 0 to 6)				
Global	−1.57 ± 1.51 (677)	72%	69%	72%
Nasal	−2.12 ± 1.84 (675)	77%	81%	76%
Ocular	−1.23 ± 1.76 (673)	56%	16%	73%
Activity	−1.65 ± 1.77 (676)	72%	75%	71%
Others	−1.43 ± 1.50 (675)	71%	67%	72%
**Global impact** (visual analog scale from 0 to 10)	−2.82 ± 3.12 (649)	65%	73%	62%
**Daytime sleepiness**				
**Epworth scale** (visual analog scale from 0 to 10)	−2.18 ± 5.01 (660)			

*MCID: minimal clinically important difference. MCID was set at −0.7 for mini-RQLQ scores and at −1 for quality of life global impact.

Regarding safety, 90 AEs were recorded in 66 patients (6.1%) during follow-up: sleepiness, fatigue, dry mouth, headache, cardiac disorders, etc. These AEs were reported by patients as the reason for premature withdrawal of treatment. At D30, 2.5% of patients declared that their tolerance towards the new H1A was worse than towards their previous treatment, versus 42.2% who declared it was better and 54.9% who reported that tolerance was the same.

### Response to treatment

**Figure 5:**
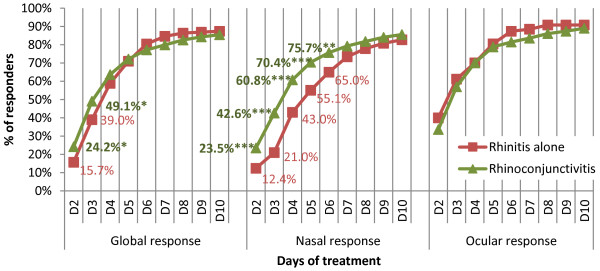
Rate of responders between D2 and D10.

The mean time to global recovery ± SD (*i.e.* to total relief of symptoms) was 6.6 days ± 3.7 (median time = 6 days). During the 10 days of treatment, the rate of responders (whose symptoms decreased to below half of their baseline level, as detailed in Methods) was 86.1%. Consequently, 13.9% of patients were considered as non-responders to treatment. The responders were distributed into early responders (78.2%) and late responders (7.9%), whether the response occurred before (and including) D6 or after D6.The course of symptom relief differed between patients suffering from rhinitis alone and those diagnosed with rhinoconjunctivitis, as shown in Figure 5. In the event of associated conjunctivitis, the rate of global response was significantly higher during the early days of treatment than for rhinitis alone (D2 and D3: p ≤ 0.02). The difference was mainly nasal symptoms, which were significantly more persistent until D6 in the event of rhinoconjunctivitis (p ≤ 0.004). Meanwhile, the frequency of ocular symptoms relief was similar between groups, but higher than 35% from D2, and reaching 90% at D10. Otherwise, there was no difference in the course of response (global, nasal and ocular) between patients with intermittent or persistent rhinitis (not shown).

According to the multivariate analysis, one factor emerged as predictive of the global response to treatment. Indeed, a patient reporting a food allergy was 4.7 fold at risk of being a late responder than a patient with no food allergy (p = 0.045). All other variables entered in the model (sex, time from the onset of rhinitis, occurrence of asthma, occurrence of conjunctivitis, periodicity of rhinitis (intermittent or persistent), previous treatment, familial history of allergy ) failed to explain the variability of the response to treatment. It should be noted that the severity of asthma (as graded in the GINA classification) was excluded from the model, as the rate of missing values was very high for this variable.

## Discussion

The present survey observed 1,080 patients with allergic rhinitis treated with second generation H1A by allergy specialists in current practice. It revealed that the main reason to opt for this new treatment was its expected efficacy, while the main reason to interrupt the previous treatment was poor efficacy.

One of the inclusion criteria specified that patients would receive second generation oral H1A in monotherapy, depending on the physician’s decision. According to ARIA guidelines, only patients with mild symptoms of allergic rhinitis should have met this criterion. However, most of the patients had moderate to severe rhinitis. This observation is in accordance with a cross-sectional prescription survey conducted in France [13] and confirmed that, in real life practice, physicians prescribe H1A as first line treatment.

In this cohort, the main compound chosen by practitioners was bilastine. Some patient characteristics differed between this cohort and that of phase III studies designed to assess the efficacy or safety of bilastine. Firstly, the current study included patients with intermittent and persistent allergic rhinitis or rhinoconjunctivitis (according to ARIA classification [5]), whereas clinical trials selectively included patients with seasonal [14,15] or perennial [16] allergic rhinitis only. Secondly, 18% of patients in this observational study exhibited mild symptoms of allergic rhinitis, while clinical trials included patients with moderate to severe symptoms only [14-16]. Thirdly, the measures of efficacy and the assessment times were not identical to those used in clinical trials. Indeed, nasal and ocular symptom scores were simplified in the current study, as compared to those used in clinical trials [14-16], due to the study design, which was observational and based solely on patient assessment during follow-up. Here, several efficacy indicators were employed: symptom score rating, quality of life rating on Likert scales and visual analog scales, relevant outside randomized clinical trials. If only because of these differences, the data from clinical trials should not be compared with those of the present study. However, the improvements of RQLQ sub-scores were comparable to those of some clinical trials [15,17]. To improve the analysis, the rate of patients reaching the MCID for quality of life scores was determined, according to methods employed by the US Agency for Healthcare Research and Quality [11]. It revealed that a majority of patients achieved MCID during follow-up, reinforcing the role of H1A in allergic rhinitis/rhinoconjunctivitis sufferers.

Whereas the estimated number of subjects required was 2,000, only 1,080 with usable data were finally included in the study. Indeed, the number of required patients had been overestimated. However, the sample size was large enough to allow descriptive analysis of the primary objective. In fact, the hypothesis chosen to estimate sample size (*i.e.* tolerance was likely to be the main reason cited to opt for a second generation H1A) was not verified. Conversely to what was assumed, the main reason for choosing a second generation H1A was its expected efficacy. This reason was far more cited than tolerance or the rapidity of action of the new compound. These data suggest that physicians consider second generation H1A as very safe, the differentiating property being efficacy. In line with this assumption, the incidence of treatment emergent adverse events for bilastine or desloratadine, measured in double-blind randomized clinical trials, was not different from that of placebo [15].

The present survey confirmed that second generation oral H1A are effective in relieving nasal and ocular symptoms in real-life conditions. The time course of the effect was quite rapid, as recovery was obtained by 6 to 7 days on average. Furthermore, symptoms were greatly reduced within the first days of treatment for most patients. Response to treatment was particularly rapid in the subgroup of patients with conjunctivitis associated with allergic rhinitis, compared to those with rhinitis alone, particularly when nasal response was examined. The response with regard to ocular symptoms was higher than that for nasal symptoms, throughout the 10-day treatment course and regardless of whether or not conjunctivitis was associated with rhinitis. On the contrary, the periodicity of the disease (persistent or intermittent) did not modify response kinetics. Evolution was comparable to that seen in randomized clinical trials [14,15]. However, due to the non comparative design of the study, we could not rule out whether symptom relief under treatment was greater than under placebo or in the absence of treatment. It should also be noted that about 33% of patients who were included and answered the first questionnaire during the medical consultation did not comply when they returned home and did not return the 10-day auto-questionnaire. However, those who did accept were fairly compliant to survey requests, as the rate of missing data was only around 10%. In addition, only 3% of patients were then lost to follow-up between D10 and D30. The rate of patients lost to follow-up did not impact the assessment of the main objective, but may have generated a bias in the analysis of response to treatment, as it cannot be excluded that patients who did not return the auto-questionnaire were poorly satisfied with the treatment. Conversely, it can be assumed that those patients who were rapidly relieved from allergic rhinitis symptoms were poorly motivated to complete the questionnaire. We are unable to discriminate between these two hypotheses. However, the profile of lost to follow-up patients was similar at baseline to that of the whole cohort (not shown).

The definition of responders was based on a 50% reduction in symptom intensity. This choice seemed relevant from a clinical view point, as the threshold was largely higher than a potential placebo effect, which has been estimated to be around 27% [18]. In accordance to the evolution of symptoms, nearly all patients who responded to treatment were classified as early responders. The analyses did not highlight any patient characteristic that could influence the response to treatment, except the occurrence of a food allergy, which was the most frequent concomitant allergy reported by patients. Associated food allergies may well represent a marker of a more severe atopic background, as shown in one study of asthma phenotype in children [19]. The multivariate regression model also showed that patients with controlled asthma were more likely to be late responders than patients without asthma. However, this trend was not statistically significant (OR = 1.9; p = 0.07).

Although they were diagnosed with rhinitis alone by the allergy specialist during the inclusion visit, 47.7% of patients reported ocular symptoms in their auto-questionnaire at D0. However, the mean score for ocular discomfort assessed by patients on the 10-point Likert scale was 2.8 in the “rhinitis alone” group, compared to 6.4 in the “rhinoconjunctivitis” group. It is likely that there was a gap between the diagnosis of conjunctivitis by physicians and the perception of ocular symptoms by patients. Consequently, it may be questionable whether comparisons between “rhinitis alone” and “rhinoconjunctivitis” groups on the basis of doctors’ assessments are relevant. However, a significant difference was noted at baseline regarding the impact of the disease on quality of life.

Although many patients responded to the 10-day treatment with second generation H1A in the studied cohort, a minority (about 14%) were classified as non-responders. Non-compliance can partially account for the lack of efficacy, as described by others [20]. However, this parameter was hardly recordable in an observational study. Treatment failure for H1A has also been reported to be more frequent in children when allergic rhinitis is caused by house dust mites, compared to pollens, *i.e.* in the event of persistent rather than intermittent rhinitis [21]. In the current study, however, the periodicity of rhinitis was not associated with the response rate.

Overall, between 8 and 9 patients out of 10 declared being satisfied with their second generation oral H1A, which significantly improved their quality of life during treatment. This ratio is high, compared to the 60% of dissatisfied allergic rhinitis patients seeking another medication to improve their health [22].

## Competing interest

Pascal Demoly received fees from Pierre Fabre Medicament France to design and coordinate the study and to analyze the results. He is also a consultant and a speaker for Stallergenes, ALK, Circassia and Chiesi and was a speaker for Merck, Astra Zeneca, Menarini and GlaxoSmithKline. Anca M. Chiriac has no conflict of interest. Benoît Berge is employed by Euraxi Pharma. Michel Rostin is employed by Pierre Fabre Médicament France.

## Authors’ contributions

PD was the medical expert involved in designing the study protocol, coordinating the study and analyzing the results. AMC contributed to data analysis and manuscript writing. BB carried out data management and statistical analysis. MR coordinated the study organization. All authors read and approved the final manuscript.

## References

[B1] Klossek JM Annesi-Maesano I Pribil C Didier A [INSTANT: national survey of allergic rhinitis in a French adult population based-sample] Presse Med 2009 38 9 1220 1229 10.1016/j.lpm.2009.05.012 19647393

[B2] Canonica GW Bousquet J Mullol J Scadding GK Virchow JC A survey of the burden of allergic rhinitis in Europe Allergy 2007 62 Suppl 85 17 25 17927674 10.1111/j.1398-9995.2007.01549.x

[B3] Ramirez LF Urbinelli R Allaert FA Demoly P Combining H1-antihistamines and nasal corticosteroids to treat allergic rhinitis in general practice Allergy 2011 66 11 1501 1502 10.1111/j.1398-9995.2011.02682.x 21883275

[B4] Simons FE Simons KJ H1 antihistamines: current status and future directions World Allergy Organ J 2008 1 9 145 155 23282578 10.1186/1939-4551-1-9-145 PMC3650962

[B5] Bousquet J Schunemann HJ Samolinski B Demoly P Baena-Cagnani CE Bachert C Bonini S Boulet LP Bousquet PJ Brozek JL Canonica GW Casale TB Cruz AA Fokkens WJ Fonseca JA van Wijk RG Grouse L Haahtela T Khaltaev N Kuna P Lockey RF Lodrup Carlsen KC Mullol J Naclerio R O'Hehir RE Ohta K Palkonen S Papadopoulos NG Passalacqua G Pawankar R Allergic Rhinitis and its Impact on Asthma (ARIA): achievements in 10 years and future needs J Allergy Clin Immunol 2012 130 5 1049 1062 10.1016/j.jaci.2012.07.053 23040884

[B6] Valovirta E Myrseth SE Palkonen S The voice of the patients: allergic rhinitis is not a trivial disease Curr Opin Allergy Clin Immunol 2008 8 1 1 9 10.1097/ACI.0b013e3282f3f42f 18188010

[B7] Demoly P Allaert FA Lecasble M Bousquet J Validation of the classification of ARIA (allergic rhinitis and its impact on asthma) Allergy 2003 58 7 672 675 10.1034/j.1398-9995.2003.t01-1-00202.x 12823130

[B8] GINA Global Strategy for Asthma Management and Prevention 2012 (update) 2012 Global Initiative for Asthma http://www.ginasthma.org/, viewed on 06/16/2014

[B9] Juniper EF Thompson AK Ferrie PJ Roberts JN Development and validation of the mini Rhinoconjunctivitis Quality of Life Questionnaire Clin Exp Allergy 2000 30 1 132 140 10.1046/j.1365-2222.2000.00668.x 10606940

[B10] Johns MW A new method for measuring daytime sleepiness: the Epworth sleepiness scale Sleep 1991 14 6 540 545 1798888 10.1093/sleep/14.6.540

[B11] Glacy J Putnam K Godfrey S Falzon L Mauger B Samson D Aronson N Treatments for Seasonal Allergic Rhinitis 2013 Rockville (MD): Agency for Healthcare Research and Quality (US) 23946962

[B12] Falliers CJ Brandon ML Buchman E Connell JT Dockhorn R Leese PT Miller J Wasserman SI Zeterberg JM Altman R Love S Samuels LL Double-blind comparison of cetirizine and placebo in the treatment of seasonal rhinitis Ann Allergy 1991 66 3 257 262 1672494

[B13] Demoly P Concas V Urbinelli R Allaert FA Spreading and impact of the World Health Organization's Allergic Rhinitis and its impact on asthma guidelines in everyday medical practice in France. Ernani survey Clin Exp Allergy 2008 38 11 1803 1807 18721255 10.1111/j.1365-2222.2008.03085.x

[B14] Kuna P Bachert C Nowacki Z van Cauwenberge P Agache I Fouquert L Roger A Sologuren A Valiente R Efficacy and safety of bilastine 20 mg compared with cetirizine 10 mg and placebo for the symptomatic treatment of seasonal allergic rhinitis: a randomized, double-blind, parallel-group study Clin Exp Allergy 2009 39 9 1338 1347 10.1111/j.1365-2222.2009.03257.x 19438584

[B15] Bachert C Kuna P Sanquer F Ivan P Dimitrov V Gorina MM van de Heyning P Loureiro A Comparison of the efficacy and safety of bilastine 20 mg vs desloratadine 5 mg in seasonal allergic rhinitis patients Allergy 2009 64 1 158 165 10.1111/j.1398-9995.2008.01813.x 19132976

[B16] Sastre J Mullol J Valero A Valiente R Efficacy and safety of bilastine 20 mg compared with cetirizine 10 mg and placebo in the treatment of perennial allergic rhinitis Curr Med Res Opin 2012 28 1 121 130 10.1185/03007995.2011.640667 22077106

[B17] Noonan MJ Raphael GD Nayak A Greos L Olufade AO Leidy NK Champan D Kramer B The health-related quality of life effects of once-daily cetirizine HCl in patients with seasonal allergic rhinitis: a randomized double-blind, placebo-controlled trial Clin Exp Allergy 2003 33 3 351 358 10.1046/j.1365-2222.2003.01596.x 12614450

[B18] Demoly P Didier A Blay F Vervloet D Devillier P Taille de l’effet thérapeutique dans les essais cliniques de la rhinite allergique saisonnière Revue Francaise D Allergologie 2011 51 2 84 94 10.1016/j.reval.2011.02.004

[B19] Just J Gouvis-Echraghi R Rouve S Wanin S Moreau D Annesi-Maesano I Two novel, severe asthma phenotypes identified during childhood using a clustering approach Eur Respir J 2012 40 1 55 60 10.1183/09031936.00123411 22267763

[B20] Koberlein J Kothe AC Schaffert C Determinants of patient compliance in allergic rhinoconjunctivitis Curr Opin Allergy Clin Immunol 2011 11 3 192 199 10.1097/ACI.0b013e3283466fcb 21467925

[B21] Turner PJ Kemp AS Allergic rhinitis in children J Paediatr Child Health 2012 48 4 302 310 10.1111/j.1440-1754.2010.01779.x 20598067

[B22] Marple BF Fornadley JA Patel AA Fineman SM Fromer L Krouse JH Lanier BQ Penna P Keys to successful management of patients with allergic rhinitis: focus on patient confidence, compliance, and satisfaction Otolaryngol Head Neck Surg 2007 136 6 Suppl S107 S124 17512862 10.1016/j.otohns.2007.02.031

